# Target selection of soluble protein complexes for structural proteomics studies

**DOI:** 10.1186/1477-5956-3-3

**Published:** 2005-05-18

**Authors:** Weiping Shen, Steven Yun, Bonny Tam, Kush Dalal, Frederic F Pio

**Affiliations:** 1 Department of Molecular Biology and Biochemistry, Simon Fraser University, 8888 University Drive, Burnaby, British Columbia, Canada, V5A 1S6

## Abstract

**Background:**

Protein expression in *E. coli *is the most commonly used system to produce protein for structural studies, because it is fast and inexpensive and can produce large quantity of proteins. However, when proteins from other species such as mammalian are produced in this system, problems of protein expression and solubility arise [1]. Structural genomics project are currently investigating proteomics pipelines that would produce sufficient quantities of recombinant proteins for structural studies of protein complexes. To investigate how the *E. coli *protein expression system could be used for this purpose, we purified apoptotic binary protein complexes formed between members of the Caspase Associated Recruitment Domain (CARD) family.

**Results:**

A combinatorial approach to the generation of protein complexes was performed between members of the CARD domain protein family that have the ability to form hetero-dimers between each other. In our method, each gene coding for a specific protein partner is cloned in pET-28b (Novagen) and PGEX2T (Amersham) expression vectors. All combinations of protein complexes are then obtained by reconstituting complexes from purified components in native conditions, after denaturation-renaturation or co-expression. Our study applied to 14 soluble CARD domain proteins revealed that co-expression studies perform better than native and denaturation-renaturation methods. In this study, we confirm existing interactions obtained *in vivo*in mammalian cells and also predict new interactions.

**Conclusion:**

The simplicity of this screening method could be easily scaled up to identify soluble protein complexes for structural genomic projects. This study reports informative statistics on the solubility of human protein complexes expressed in *E.coli *belonging to the human CARD protein family.

## Background

The aim of structural proteomics is to experimentally derive the 3-dimensional structure of all proteins in genomes through determination of the 3D structure of sufficient members in a protein family such that the remaining structures can be predicted accurately through computational approaches [2]. The success rates in studies so far, mainly applied to prokaryotes, are still low. Effectively, 1 to 5% of structures are solved experimentally from a few thousand of Open Reading Frame (ORF) tested per study. The reasons for this limitation are primarily due to the fact that many proteins are insoluble when expressed in a heterologous system or when purified and concentrated at the levels necessary for crystallization studies. The success rate is even lower when applied to eukaryotes, where for example, it was shown recently that in *C. elegans *only 20% of the ORF cloned produced soluble recombinant protein suitable for structural studies. This rate is significantly lower than that of structural genomics studying prokaryotics ORF [3]. In these studies, a tremendous amount of effort has been spent to improve protein solubility by switching the expression system, changing the fusion tags, replacing the expression host, generating a new structural variant by modifying the protein through genetic engineering including mutagenesis [4] and computational approaches, and finally by performing crystallization studies on a protein orthologs to the initial protein candidate [2,5]. However, full automation of these approaches needs to be improved and new technologies are constantly being implemented in the pipelines to increase our success rates in structure determination. An even more challenging and important task for structural proteomics studies is to determine the 3D structure of all the protein complexes of an organism. Most proteins do not work as a monomer, but interact with other proteins to perform their functions in the form of stable or transient complexes. In addition, the knowledge of these interactions is of fundamental importance, since the genome complexity of an organism is not simply related to its number of genes but rather is more directly related to the complexity of its protein-protein interaction networks. Full automation processes are still difficult because the proteomics pipeline to obtain sufficient quantities of soluble protein complexes has to allow for the possibility of different purifications scenarios for each protein complex. Furthermore, protein complexes are generally difficult to reconstitute from individual protein components and usually it is almost impossible to purify sufficient quantities of *in vivo *protein complexes for structural studies. Finally, interactions between proteins in cells are effectively dynamic with a wide range of affinities. The half-life of protein complexes is quite diversified from stable complexes to transient interactions and can be dependent on post-translation modifications. The post-translational modification may not be reproduced during *in vitro *experiments or are inherently not suitable for crystallization studies since highly stable systems are necessary for successful crystallographic studies [6-8].

Three steps are needed to obtain sufficient protein complex *in vitro *to perform structural studies of protein complexes from individual components. (i) Produce soluble protein, (ii) make a soluble complex, and (iii) generate high-concentration and highly purified protein complexes to tackle the crystallization project. In this study, we focus on the first step, that of obtaining soluble protein complexes. A few structural genomics initiatives are currently trying to develop proteomics pipelines that includes the production in *E.coli *of recombinant protein complexes [9]. We tried to identify an ideal protein family that could be used for this purpose. It should be a small soluble domain that can perform simple protein complexes and from which many protein complexes have been identified *in vivo*. As a result we choose the human CARD protein family. This protein family belongs to the death domain super-family consisting of the Death Domain (DD), Death Effecter Domain (DED) and the Pyrin, AIM (Absent-in-melanoma), ASC, Apoptosis-associated speck-like protein containing a caspase recruitment domain CARD, and Death-Domain (DD)-like (PAAD/DAPIN/PYRIN) domain subfamilies. The CARD domain family contains 35 members that are involved in apoptosis and share a domain of 85 amino-acids folded into a 6 helix bundle. The domain is a recruitment domain that has the ability to form hetero-dimers between members of the same family providing a structural framework to complete the apoptotic cascade leading to caspase activation and cell death. To date, two different structures of death domain complex have been solved by x-ray crystallography represented by the structure of the human CARD domains of Apoptotic Protein Activating Factor-1 (APAF-1) with caspase-9[10], and the death domain of Pelle with Tube in Drosophillia[11]. Structural analysis of these two different protein complexes revealed different modes of protein-protein recognition while similar domains are involved in protein-protein interaction.

In this work, the first step of a structural proteomic project to obtain soluble protein complexes from the CARD domain is reported. From 25 CARD proteins cloned and expressed in *E.coli*, 14 different CARD soluble proteins were obtained. Protein purification of CARD recombinant proteins were performed and binary protein complexes were reconstituted systematically from purified individual components under native, denaturation-renaturation conditions and by co-expressing two different CARD proteins simultaneously in *E. coli *to identify stable protein-protein interactions.

## Results

### Expression and solubility

To study the interaction between CARD-containing proteins, 14 CARD recombinant proteins were subjected to three different binding assays. First, all recombinant clones were characterized for their expression and solubility in *E. coli*. Comparison of the total cell lysate and soluble protein fractions revealed that most of the CARD proteins were expressed at medium or high levels (2–5 mg/liter). Only pET constructs (that add a poly-histidine to the N-terminus of the expressed protein of interest) containing the CARD domains of the cellular homolog of the equine herpesvirus-2 E10 gene containing an amino-terminal caspase recruitment domain (BCL10) and the apoptotic protein containing a CARD domain (CLAN) (pET-28b-BCL10 and pET-28b-CLAN) were producing a recombinant protein expressed at low level (less than 1 mg/ml). Protein expression was induced at two different temperatures (37°C and 30°C) and two different IPTG concentrations (1 mM and 0.1 mM) to find the optimal solubilization conditions.

Three pET-28b-constructs of the, CARD only protein (COP), NucleOLar protein 3 (NOL3), and the Apoptosis-associated Speck-like protein containing a CARD (ASC) (pET-28b-COP, pET-28b-NOL3, and pET-28b-ASC) produced in *E.coli *recombinant proteins were insoluble. Although some improvement of solubility was seen after induction at 30°C, we classified them as "not soluble" because of low yield (below 1 mg/L of culture). These insoluble proteins were purified from *E. coli *under denaturing conditions and then renatured back to the native state. The status of refolding was confirmed by circular dichroism (CD) experiments (data not shown).

### Protein purification and direct native binding assay

The 14 cloned, CARD-containing proteins were purified on a larger scale and their optimal solubilization conditions were determined empirically. If insufficient protein purity (<90%) was obtained after his-tag affinity purification, some of the proteins were further subjected to ion-exchange chromatography. A total of 91 samples were initially set up for direct binding under native conditions. A quick way to observe any binding was to run these samples on a continuous non-denaturing polyacrylamide gel, along with the individual proteins as a control. Any shift in the electrophoretic mobility relative to that of the control lanes would indicate positive binding. This approach resulted in the identification of one direct binding and 3 indirect bindings (See additional file 1, Figure 1). Only one sample, run on a non-denaturating polyacrylamide gel from a mixture of Apaf-1 and caspase-9, showed a shifted band relative to Apaf-1 and caspase-9 control bands. In 3 other samples of the apoptotic protein containing a nucleotide binding domain and a CARD domain (NAC) and ASC, CARD-9 and BCL-10, ASC and BCL-10, we observed a decrease in the intensity of the stained bands for the protein mixture sample lane corresponding to the individual protein, but we did not detect any shifted band for the protein complex. We suggest that in these assays binding occurred because an equal amount of protein was loaded in the control lanes as in the protein mixture sample lanes, but the total charge of the protein complex is such that the complex cannot migrate into the gel during the electrophoresis. Consequently, the protein complex is not detected by the staining procedure. In addition, the two protein-protein interactions between NAC and ASC, CARD-9 and BCL-10 were confirmed by other investigators [12,13] while the binding between ASC and BCL-10 has not yet been determined experimentally. There may also be additional bindings that were not detected because of the limited sensitivity of Coomassie blue staining.

**Figure 1:**
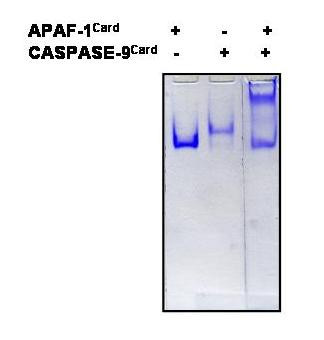
**Native binding assay between Apaf-1 and Caspase-9 detected by PAGE**. Binding reactions were performed using an equal amount of putative interacting histidine-tagged native recombinant proteins and incubation overnight in the binding buffer containing 50 mM Tris, 100 mM NaCl, pH 8.0. The binding was analyzed by a continuous Poly-Acrylamide Gel Electrophoresis (PAGE) (10%) followed by Coomassie Blue staining.

### Induced denaturation-renaturation binding assay

**Figure 2:**
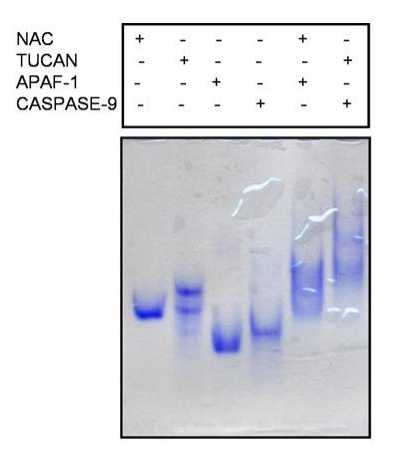
**Denaturation-renaturation binding assay between NAC and APAF-1, TUCAN and CASPASE-9 detected by PAGE**. Continuous PAGE analyses of CARD-containing proteins. An equal amount of proteins were incubated in a dialysis bag and dialyzed sequentially in a denaturation buffer, renaturation buffer and binding buffer (see material and methods). The protein complex formation was visualized by non-denaturating PAGE.

To identify any additional bindings, we decided to denature the protein mixture first, and then refold both proteins in order to obtain the protein complex. This approach resulted in the identification of a total of 5 bindings including the one observed from the native binding assay with Apaf-1 and caspase-9 (See additional file 2). Non- denaturating polyacrylamide gel electrophoresis revealed bindings within the following proteins: The Apoptotic Protein Activating Factor-1 (Apaf-1) and caspase-9, NAC and Apaf-1, the Tumor Up-regulated CARD-containing Antagonist of caspase-Nine (TUCAN) and caspase-9, NAC and CLAN, and CARD-9 and CLAN (Figure 2). NAC and Apaf-1 have been previously shown to bind to each other by immunoprecipitation [14]. Bindings were also reported for TUCAN and caspase-9 [15], and NAC and CLAN [16], which is consistent with our findings. In addition, one additional binding observed between CLAN and CARD-9 was newly identified.

### Co-expression analysis

**Figure 3:**
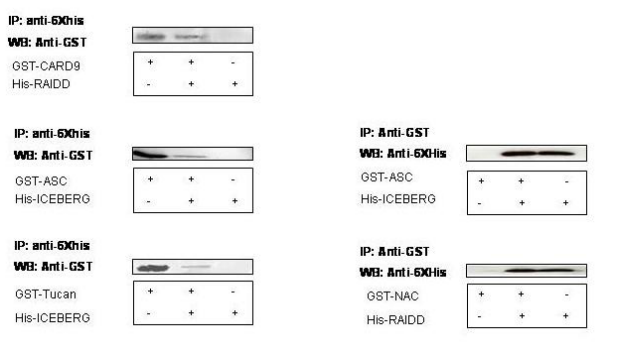
**Immunoprecipitation of putative CARD binary complexes obtained from co-expression assay**. Protein lysates obtained from the co-expressed *E. coli *putative CARD complexes were immunoprecipitated with anti-6XHis or anti-GST antibody. The precipitated protein samples (lane 3) were loaded onto a SDS-PAGE along with the individually-expressed protein lysates (lane 1 and 3). The protein samples from the gel were transferred onto Hybond-ECL (Amersham Biosciences). The immunoprecipitated protein complexes were detected with anti-GST and anti-6XHis antibody.

In this study, the interaction of 14 CARD-containing proteins was investigated by co-expression in *E. coli *using a two-vector expression system with two different antibiotic resistances. The chosen expression vectors were pET-28b and pGEX-2T (that add a glutathione S-Transferase at the N-terminus of the expressed protein) with kanamycin and ampicillin resistances, respectively. Since only the protein expressed from pET-28b carries an N-terminal histidine-tag, retention of the other protein on a Ni-NTA affinity column depends on its interaction with the pET-28b expressed protein. In order to prevent any possible false positive bindings, pGEX-2T proteins were expressed alone and were also subjected to Ni-NTA affinity chromatography. In addition, each CARD protein was cloned in both vectors to perform the assay in both directions. A total of 156 co-expressions were performed and analyzed by SDS-PAGE. We obtained 12 positive bindings (See additional file 3). Among them, NAC, CARD-9, and the Receptor-Interacting Protein (RIP)-Associated protein with a Death domain (RAIDD) could form homodimers, regardless of which vector was used in the pull down assay (Figure 3). All bindings observed in the previous two assays were also detected. The previously reported binding of CLAN and ASC [17] was also confirmed. However, there were some reported bindings that we did not obtain in this study including: The binding of CLAN and BCL-10 [16] CARD-9 and BCL-10 [13], TUCAN and the caspase-1 inhibitor (ICEBERG) [18], TUCAN and COP [18], and COP and RICK [19]. In addition, some new bindings were observed between CARD-9 and RAIDD, CARD-9 and CLAN, RAIDD and NAC, ASC and ICEBERG

**Figure 4:**
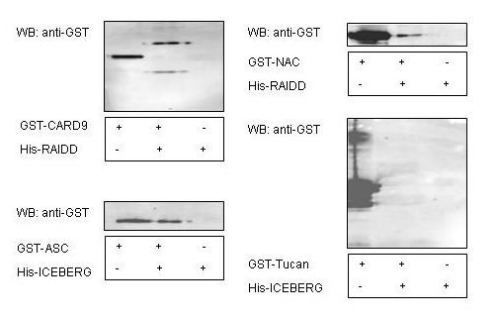
**Binary complex formation of CARD protein complex in rabbit reticulocyte lysates**. Equal amounts of in vitro expressed proteins were incubated in rabbit reticulocyte lysates at 4°C for 1 hour. After incubation, protein and complexes were immunoprecipitated with anti-6XHis antibody. The precipitated samples (lane 2) together with the individually-expressed proteins (lane 1 and 3) were detected by western blot using anti-GST antibody.

In order to determine whether these *in vitro *bindings from co-expression studies also occur in mammalian system, equal amounts of *E. coli *recombinant proteins were incubated in rabbit reticulocyte lysate (Figure 4), followed by immuno-precipitation and western blotting. As expected, we confirmed protein complex formation in the mammalian system among the newly identified protein complexes by co-expression. One interesting observation is that the co-expression of the pET-28b-ASC and pGEX-2T-ICEBERG construct, leads to a soluble protein complex, while the pET-28b-ASC when expressed individually produced a recombinant protein considered insoluble.

## Discussion

**Figure 5:**
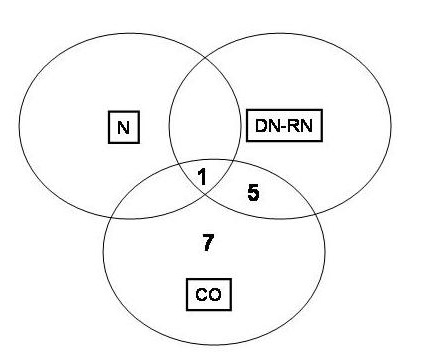
**Venn diagram representing the overlap between the 3 binding assays tested**. N, represent native, CO, co-expression and DN-RN DeNaturation-ReNaturation assay.

In order to develop a low cost, simple and reliable system, which may be used to generate *in vitro *sufficient quantity of protein complexes for protein structure determination, we compared three *in vitro *binding assay systems: native binding assay, denaturation-renaturation binding assay, and co-expression binding assay. While these three different systems can be easily scaled up to generate large quantities of protein complex at very low cost, their accuracy in reproducing known CARD-CARD interactions described in the previous literature were quite different (Figure 5). According to previous studies, seven CARD protein complexes were reported within our 14 CARD domain members ([12-17,20]). Co-expression assay reproduced five of them with the highest accuracy of 71.4%. Induced denaturation-renaturation binding assay was able to generate four complexes from seven published complexes (57%). Native binding assay detected three complexes from the seven identified complexes with an accuracy of 42.9%. In addition, these three *in vitro *assays also identified a total of four new CARD protein complexes. Co-expression studies identified all four new complexes and the other two methods identified one complex each. Our results from rabbit reticulocyte lysate incubation study indicated that CARD protein complexes obtained between RAIDD and NAC, ASC and ICEBERG could be formed in rabbit reticulocyte mammalian system. A protein-protein interaction detected between CARD9 and RAIDD was also identified, but the identified proteins had different sizes compared to the *E.coli *recombinant proteins not incubated in the rabbit reticulocyte lysate. These data might suggest that the differences observed in the electrophoretic mobility of the interacting proteins could be accounted for by aggregation, post-translational modification or assisted folding. Such events would occur during their incubation in the mammalian lysate. It is not surprising that the CARD domain of ASC and ICEBERG could interact with each other in our assay since both CARD domains have been shown by others to bind to a common protein procaspase-1. Effectively, recent studies [21] demonstrated that ICEBERG binds to procaspase-1 through CARD- CARD interaction, which prevented procaspase-1 association and activation with the NF-kappaB-activating and cell death-inducing kinase RIP2. As a result, ICEBERG inhibits the processing of procaspase-1 essential for the activation of caspase-1. In addition, ASC also interacts with the CARD domain of procaspase-1[22]. Functional outcome of this interaction is dependent on the expression level of ASC in cells, since ASC enhances caspase-1 secretion into the cell culture at low concentration, but suppresses it at high concentration. Further studies will need to be performed to assess the functional significance of the interaction between ASC and ICEBERG. It is also quite striking that the newly identified protein complex obtained between NAC [14] and RAIDD [23] are among two proteins targets of the stress-induced apoptosis pathway, but previously shown to be involved at different point of the mitochondrial intrinsic pathway. RAIDD participates in the activation of caspase-2 by forming a multiprotein complex named P53-Induced protein with a Death Domain protein complex (PIDDosome) [24] that contain PIDD, RAIDD, and caspase-2. On the contrary NAC increases the processing of caspase-9 by binding to Apaf-1 in a multiprotein complex named the apoptosome consisting of apaf-1 and procaspase-9. So far, there is no evidence or publications to indicate a protein interaction between the CARD domain of RAIDD and NAC. Further functional studies need to be performed to assess the significance of this protein complex in apoptosis. Co-incubation between CARD9 and CLAN in a mammalian system failed to demonstrate interaction between the CARD domains of these two proteins. The *in vitro *protein complex formed between the CARD domains of CARD9 and CLAN in the co-expression assay could be an artificial or a newly identified complex. Further investigation, such as co-expression of these two proteins in mammalian system, should be performed to confirm these results.

In this study, co-expression binding assay performed the best compared to the other 2 approaches tested. It was also the simplest assay system since it did not require prior purification of the binding partners before complex formation. In addition, we observed that some proteins were not soluble when expressed alone, but could form a soluble protein complex in co-expression experiments. For example, the CARD domain of ASC protein is barely soluble when expressed alone in *E. coli*, but its complex with the CARD domain of ICEBERG in co-expression assay is totally soluble.

Both, native binding assay and induced denaturation-renaturation binding assay were more sensitive to sample preparation. For the native assay, among most of the protein pairs tested the binding was not detected even when both proteins were well folded and stable. This could indicate that the binding conditions needs to be optimized for each complex or that assisted folding and post-translational modifications are necessary for optimal binding. For the induced denaturation – renaturation binding assay, since the co-incubated pairs of proteins are denaturated then renaturated in the same conditions to form complexes, those conditions should be such that some potential protein complex could not be formed between some of the pairs tested in this study [25,26]. This may be the reason why three out of seven published CARD-CARD complexes could not be reproduced in this assay.

## Conclusion

Our experimental results indicate that the co-expression system is recommended as the first choice for structural study of protein-protein complexes. This binding assay system is simple, inexpensive and can be easily automated for structural proteomics studies.

## Methods

### Cloning

EST clones encoding CARD domain were searched in the NCBI database . 14 EST spanning the CARD domain were found and used as templates for PCR amplification. The CARD domains were amplified by PCR and cloned into the expression vector pET or pGEX to obtain the following CARD-containing proteins: NAC (residues 1373–1473), TUCAN (residues 341–431), Apaf-1 (residues 1–108), caspase-9 (residues 1–111), CARD9 (residues 14–100), BCL10 (residues 1–104), RAIDD (residues 9–95), COP (residues 1–98), NOL3 (residues 1–97), CLAN (residues 1–87), RICK (residues 417–540), ICEBERG (residues 1–90), CARD10 (residues 31–117), and ASC (residues 92–195). The individual CARD domains (except RICK) were cloned into the Nde1-BamH1 and BamH1-EcoR1 restriction sites of pET-28b (Novagen) and pGEX-2T vectors, respectively. Since RICK had an internal BamH1 site in its sequence we cloned it into Nde1-EcoR1 sites of the pET28b vector.

### Protein expression, solubility, and purification

pET-28b clones were transformed into *E.coli *BL21(DE3) (Novagen) and grown on petri dishes containing 50 μg/ml kanamycin. Cultures were grown from single colonies in 25 ml LB medium supplemented with kanamycin (40 μg/ml) at 37°C. His-tagged proteins were induced at an OD_600 _of 0.6 by the addition of IPTG to a final concentration of 1mM. After an additional 4 hours of growth at 30°C or 37°C, 2x1 mL aliquots of the culture were centrifuged to obtain cell pellets (5 min at 13000 rpm). One of the cell pellets were re-suspended in denaturing buffer and kept as the whole cell fraction. The other fractions were re-suspended in native lysis buffer (50 mM NaH_2_PO_4_, 300 mM NaCl, 10 mM imidazole), sonicated and centrifuged (5 min at 13000 rpm) in Eppendorf tubes. The resulting supernatant representing the soluble protein fraction was compared against the whole cell fraction by SDS-PAGE to determine the size, and the relative expression and solubility levels of each protein.

### Binding assay – native condition

For this assay the 14 pET-28b constructs were transformed into *E. coli *BL21 (DE3). Transformed cloned were inoculated into a 50 ml LB for overnight preculture. The non-saturated bacterial cultures were further diluted into 1 Liter of LB medium for protein expression. The cultures were grown at 37°C with shaking until an A_600 _of 0.6 was reached. Protein expression was induced by the addition of IPTG to a final concentration of 1 mM and the induction was allowed to take place for an additional 4 hours. The induced bacteria were collected by centrifugation (20 min at 4000 rpm). Purification was carried out by either native or denaturing conditions depending on the solubility status of the individual proteins. For purification under native conditions, the cells were resuspended in 15 ml 50 mM NaH_2_PO_4_, 300 mM NaCl, 10 mM imidazole, 5 mM β-mercaptoethanol, and 0.1 mM PMSF at pH 8.0. The cells were lysed with a french pressure cell and centrifuged for 30 min at 4°C at 18,000 rpm. Ni-NTA superflow (Qiagen) was used in the His-tag purification of the induced protein. 1 ml 50% Ni-NTA slurry was used to pack the chromatography column. The purification of 6xHis-tagged protein was performed in this column using a 10 to 250 mM imidazole gradient. To facilitate the purification process, the columns were centrifuged for 1 min at 4°C at 800 rpm for the washing and elution steps. The purified proteins were then dialyzed against binding buffer containing 50 mM Tris, and 100 mM NaCl at pH 8.0. For purification under denaturing conditions, the cells were re-suspended in 15 ml of 100 mM NaH_2_PO_4_, 10 mM Tris-HCl, 8 M urea, pH 8.0. After loading the lysate onto the column the renaturation was performed using a linear 6 M – 1 M urea gradient in 50 mM Tris, 100 mM NaCl, 5% glycerol, 1 mM EGTA, 0.1 mM PMSF, 2 mM MgCl_2_, 14.4 mM β-mercaptoethanol, pH 8.0. Elution of the bound proteins was performed using a pH gradient from pH 8.0 to pH 4.5. After elution of the re-natured proteins the protein solution were dialyzed against a binding buffer containing 50 mM Tris, 100 mM NaCl, pH 8.0. An equal amount of the purified proteins (91 combinations) were incubated overnight at 4°C and the binding was analyzed by continuous non-denaturing polyacrylamide gel electrophoresis (10% PAGE) followed by Coomassie blue staining.

### Binding assay – induced denaturation-renaturation

The purification of the 14 recombinant proteins were performed as described previously. An equal amount of the purified proteins (91 combinations) were dialyzed in a micro dialysis bag (100 μL of total volume used) against 8 M urea, 1 mM EGTA, 0.1 mM PMSF, 50 mM Tris, 100 mM NaCl, 14.4 mM β-mercaptoethanol, pH 8.0. The denatured proteins were then renaturated by dialyzing against a renaturation buffer containing 2 M urea, 1 mM EGTA, 0.1 mM PMSF, 50 mM Tris, 100 mM NaCl, 2 mM MgCl_2_, 5% glycerol, 14.4 mM β-mercaptoethanol. The protein complexes were detected by continuous non-denaturing polyacrylamide gel electrophoresis (10%) followed by Coomassie blue staining.

### Binding assay – co-expression in *E. coli*

A mixture of 10 ng of pET-28b and pGEX-2T constructs (156 combinations) were transformed into *E. coli *BL21(DE3) and grown on petri plates containing 40 μg/ml kanamycin with 50 μg/ml ampicillin. Cells from 5 ml overnight cultures grown at 37°C were used to inoculate 100 ml LB medium containing 40 μg/ml kanamycin and 50 μg/ml ampicillin. Protein expression was induced at an OD_600 _of 0.6 with the addition of 1 mM of IPTG. After an additional 4 hours of growth at 37°C, the cells were collected by centrifugation (10 min at 4000 rpm). The induced cells were then re-suspended in 50 mM Tris (pH 8.0), 100 mM NaCl, 5 mM β-mercaptoethanol, 0.1 mM PMSF and lysis were performed with a french pressure cell. Cell lysate were centrifuged 30 min at 4°C at 25,000 g. Ni-NTA superflow (Qiagen) affinity gel was used for the His-tag purification of the complex. Elution of the proteins was performed using a 20 to 250 mM imidazole gradient. After washing extensively, the bound proteins were eluted in 500 μL aliquots. The Ni-NTA purified co-proteins and protein complexes were loaded on SDS-PAGE and stained with silver nitrate.

### Binding assay in rabbit reticulocyte lysate

In order to verify if the positive *in vitro *interactions obtained by co-expressing both CARD protein partners in *E. coli *were also able to form protein complex in a mammalian system, equal amounts of the putative interacting proteins were incubated in 50 μL of Rabbit Reticulocyte Lysate (Promega) at 4°C for 1 hour.

The co-expressed proteins or protein complexes and the binding products in Rabbit Reticulocyte Lysate (Promega) were immunoprecipitated with anti-6xHis or anti-GST antibody. Protein-protein interactions were detected by SDS-PAGE, western blotting and anti-GST or anti-6xHIS.

## List of abbreviations

CARD: Caspase Associated Recruitment Domain, ORF: Open Reading Frame, PAAD/DAPIN/PYRIN: Pyrin, AIM (Absent-in-melanoma), ASC, Apoptosis-associated speck-like protein containing a caspase recruitment domain CARD, and Death-Domain (DD)-like/ Domain, ApoPtosis, INterferon response/Pyrin, ORF: Open Reading Frame, CD: Circular Dichroism, APAF-1: Apoptotic Protein Activating Factor-1, ICEBERG: caspase-1 inhibitor, ASC: Apoptosis-associated Speck-like protein containing a CARD, NAC: apoptotic protein containing a nucleotide binding domain and a CARD domain, RAIDD: receptor-interacting protein (RIP)-Associated ICH-1/CED-3-homologous protein with a Death domain, DD: Death domain, DED: Death effector domain, PIDD: P53-Induced protein with a Death Domain, TUCAN: Tumor Up-regulated CARD-containing Antagonist of caspase-Nine, BCl10: cellular homolog of the equine herpesvirus-2 E10 gene containing an amino-terminal caspase recruitment domain, COP: CARD Only Protein, NOL3: NucleOLar protein 3 or Apoptosis Repressor with CARD domain (ARC), CLAN: apoptotic protein containing a CARD domain, a Leucine rich repeat domain (LRR) and a NACHT domain (initially found in the genes NAip, Ciita, Het-e and Tp1), RICK: RIP-like interacting CLARP kinase, IPTG: Isopropyl-βD-thiogalactopyranoside, LB: Luria Broth medium, PMSF: phenylmethylsulfonyl fluoride SDS-PAGE: Sodium Dodecyl Sulfate Polyacrylamide Gel Electrophoresis.

## Authors' contributions

WS, performed immunoprecipitation, some co-expression assays and western blotting studies and wrote part of the manuscript, SY performed all native, renaturated and co-expression binding assays, BT was involved in cloning and immunoprecipitation experiments, FP provided guidance during this project and wrote the manuscript

## Supplementary Material

Additional File 1 Native binding assays of CARD proteins complexes (ppt). Click here for file

Additional File 2 Denaturation-renaturation binding assays of CARD protein complexes (ppt). Click here for file

Additional File 3 Co-expression binding assays of CARD protein complexes (ppt). Click here for file
