# Supplementary material for: Target selection of soluble protein complexes for structural proteomics studies
Source: Proteome Sci. 2005 May 18;3:3. doi: 10.1186/1477-5956-3-3 (PMC1156946; doi:10.1186/1477-5956-3-3)
Supplement: Additional File 1 — Native binding assays of CARD proteins complexes (ppt). [file 1477-5956-3-3-S1.ppt]

## Slide 1
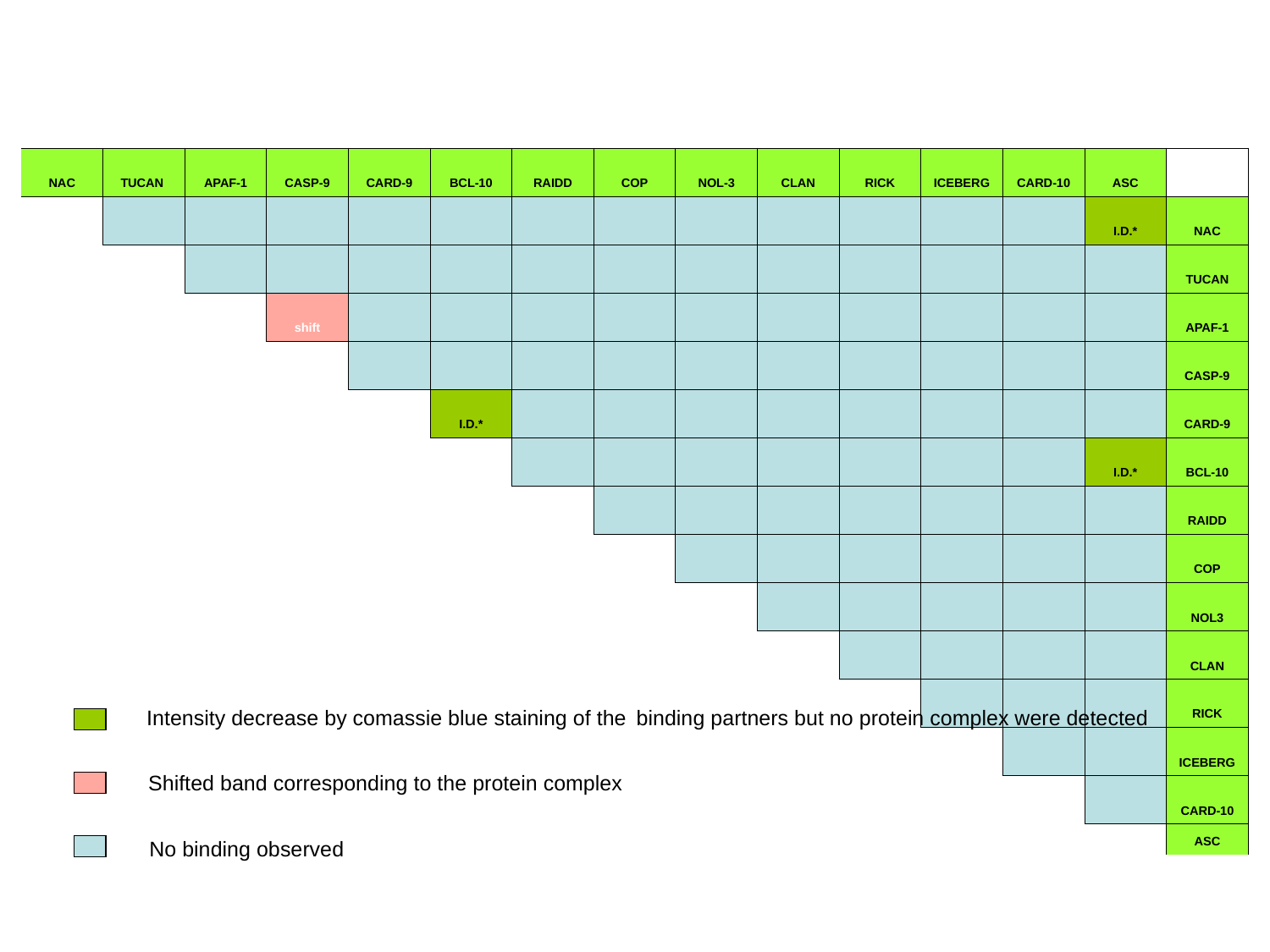

| NAC | TUCAN | APAF-1 | CASP-9 | CARD-9 | BCL-10 | RAIDD | COP | NOL-3 | CLAN | RICK | ICEBERG | CARD-10 | ASC | |
| --- | --- | --- | --- | --- | --- | --- | --- | --- | --- | --- | --- | --- | --- | --- |
| | | | | | | | | | | | | | I.D.\* | NAC |
| | | | | | | | | | | | | | | TUCAN |
| | | | shift | | | | | | | | | | | APAF-1 |
| | | | | | | | | | | | | | | CASP-9 |
| | | | | | I.D.\* | | | | | | | | | CARD-9 |
| | | | | | | | | | | | | | I.D.\* | BCL-10 |
| | | | | | | | | | | | | | | RAIDD |
| | | | | | | | | | | | | | | COP |
| | | | | | | | | | | | | | | NOL3 |
| | | | | | | | | | | | | | | CLAN |
| | | | | | | | | | | | | | | RICK |
| | | | | | | | | | | | | | | ICEBERG |
| | | | | | | | | | | | | | | CARD-10 |
| | | | | | | | | | | | | | | ASC |
Intensity decrease by comassie blue staining of the binding partners but no protein complex were detected
Shifted band corresponding to the protein complex
No binding observed
